# Modified Star Excursion Balance Testing at 12 Months After Anterior Cruciate Ligament Reconstruction: Is There a Difference Between Quadriceps or Hamstring Tendon Autografts?

**DOI:** 10.1177/23259671251331044

**Published:** 2025-04-21

**Authors:** Julian Röhm, Haydn J. Klemm, Lachlan M. Batty, Jodie A. McClelland, Brian M. Devitt, Timothy S. Whitehead, Kate E. Webster, Julian A. Feller

**Affiliations:** * OrthoSport Victoria Research Unit, Melbourne, Victoria, Australia; † School of Allied Health, Human Services and Sport, La Trobe University, Melbourne, Victoria, Australia; § Department of Orthopaedic Surgery, Western Health, Melbourne, Victoria, Australia; ‖ School of Health and Human Performance, Dublin City University, Dublin, Ireland; Investigation performed at OrthoSport Victoria Research Unit, Melbourne, Victoria, Australia

**Keywords:** modified Star Excursion Balance Test (MSEBT), Y Balance Test (YBT), anterior cruciate ligament (ACL), quadriceps tendon graft, return to sport (RTS)

## Abstract

**Background:**

Quadriceps tendon (QT) autograft has emerged as an increasingly popular graft for anterior cruciate ligament reconstruction (ACLR). The modified Star Excursion Balance Test (MSEBT) measures dynamic balance and is frequently used in evaluating preparedness to return to sport as part of return-to-sport test batteries. There is limited information available about the MSEBT performance of patients who have undergone ACLR with QT autograft.

**Hypothesis/Purpose:**

The purpose was to compare the MSEBT performance at 12 months after primary ACLR of patients with QT autografts with the performance of patients with hamstring tendon (HS) autografts. It was hypothesized that there would be a difference in the 2 groups due to harvest from either an extensor or a flexor of the knee joint.

**Study Design:**

Cohort study; Level of evidence, 3.

**Methods:**

The cohort consisted of 132 patients (44 patients with QT, 88 patients with HS) who had undergone primary ACLR with either a QT or HS autograft, were <30 years of age at the time of surgery, and had participated in sports regularly before injury. Patients with contralateral anterior cruciate ligament injury or an additional lateral extra-articular tenodesis were excluded. The mean age of the patients was 22.1 years, and 18% were female. The anterior reach, posterolateral reach, and posteromedial reach on the MSEBT were recorded at 12 months postoperatively and normalized to leg length. The limb symmetry index (LSI) and the composite score (CS) were calculated for each measurement. Patient-reported outcome measures were also collected.

**Results:**

There were no significant differences between the mean LSI and the CS of the QT and HS groups for any reach direction of the MSEBT (LSI: QT = 99.9 and HS = 98.9 for anterior reach, QT = 100.9 and HS = 100.2 for posterolateral reach; QT = 101.1 and HS = 100.8 for posteromedial reach, CS: QT = 96.6 and HS = 96.9). Patient-reported outcome measures also showed no significant difference.

**Conclusion:**

There were no differences in symmetry between QT and HS grafts in MSEBT performance at 12 months, with both patient groups having >98% limb symmetry in each reach direction.

The incidence of both anterior cruciate ligament reconstruction (ACLR) and anterior cruciate ligament (ACL) graft rupture is rising, especially in the adolescent population.^16,23,33^ In light of this, a number of return-to-sport (RTS) programs have been developed.^9,21,30^ Functional performance tests are often included. An important aspect of lower limb function is dynamic balance, which has previously been assessed using the Star Excursion Balance Test (SEBT).^14,20^ To improve the reliability and field practicability of the SEBT, the modified SEBT (MSEBT) and the Y Balance Test (YBT) were developed as simplified versions. They use only 3 reach directions (anterior, posterolateral, and posteromedial) compared with 8 with the original SEBT.^13,18,19,25^ Both tests assume that the distance of lower extremity reach is indicative of dynamic balance in the standing limb and have been investigated as a method to assess dynamic postural-control deficits.^10,19^ Unlike the MSEBT, the YBT uses a commercially available test kit.

For both tests, different thresholds for asymmetry and associated risk of injury in the future have been proposed. Several authors have suggested that a reduced limb symmetry index (LSI) for anterior reach in dynamic balance tests may be a potential risk factor for further injury to the lower extremity,^3,11,12,20,26^ and it has been recommended that an LSI >90% for functional balance and hop tests needs to be achieved for a safe RTS.^22^ Furthermore, Garrison et al^8^ concluded that a >4-cm anterior reach deficit at 12 weeks identifies those participants who may not achieve a 90% LSI on hop measures at the time of RTS. However, performance on dynamic balance tests is influenced by age, sex, and the sport played, and appropriate individual thresholds should be used when interpreting the results.^18^

Several studies have reported investigating these dynamic balance tests after ACLR with either hamstring tendon (HS) autograft or patellar tendon (PT) autograft.^6-8,27^ Sugimoto et al^27^ compared the effect of different graft types for ACLR on postoperative performance in balance and hop tests and described a significant deficit in the anterior reach of YBT in patients after ACLR with PT autograft at 6 to 9 months postoperatively, which may reflect a deficiency in function of the extensor mechanism of the knee. In the same study, there was no difference in the posteromedial and posterolateral reaches for PT, HS, and iliotibial band autografts.

Quadriceps tendon (QT) autograft has emerged as an increasingly popular graft for ACLR and has been shown to have at least equivalent results to ACLR with HS autograft.^15,17,28,34^ However, there is limited information about the performance on balance tests of patients who have undergone ACLR with QT autograft.^24,32^ As such, questions remain on whether harvest of the QT may affect function of the extensor mechanism in a similar way to when PT autograft is used. Therefore, the aim of this study was to compare the MSEBT performance in patients who had an ACLR with QT autograft with the performance of those who had an ACLR with an HS autograft at 12 months postoperatively. We hypothesized that there would be a difference in the 2 groups due to harvest from either an extensor or a flexor of the knee joint.

## Methods

### Ethics Approval

Human research ethics committee approval was obtained for this study as part of a longitudinal study investigating a cohort of patients with ACL injuries.

### Patient Population and Study Setting

**Figure 1.**
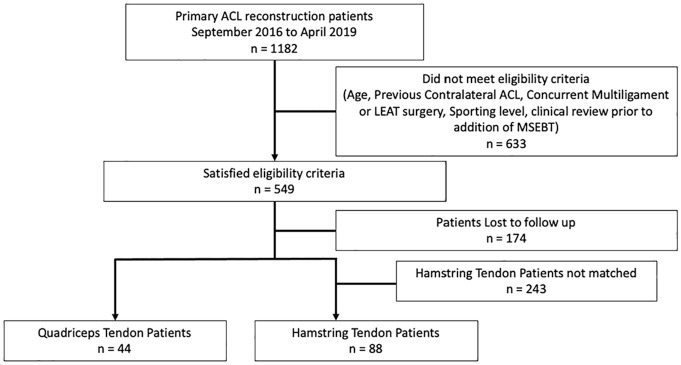
Flowchart of study patients. ACL, anterior cruciate ligament; LEAT, lateral extra-articular tenodesis; MSEBT, modified Star Excursion Balance Test.

This sex-matched cohort study was a retrospective analysis of prospectively collected data from patients who had undergone ACLR between September 2016 and April 2019 in a private orthopaedic clinic in Melbourne, Australia. The inclusion criteria for the study were age <30 years at the time of surgery and a primary ACLR with either QT or HS autograft. Patients were excluded if they had a previous contralateral or ipsilateral ACL injury or an injury of the collateral ligaments or multiligament injury that required surgical intervention. Further exclusion criteria were an additional lateral extra-articular tenodesis at the time of ACLR and a preinjury level of sport participation documented by the patient as either “nonsporting” or “sports sometimes.” Meniscal and chondral lesions were not exclusion criteria. Each patient with a QT autograft was matched to 2 patients of the same sex with an HS autograft. The final cohort included 44 QT patients and 88 HS patients. The MSEBT was performed at the 12-month postoperative visit. A flowchart of the study patients is shown in Figure 1.

### Surgical Technique and Rehabilitation

All ACLR procedures were performed by 1 of 4 experienced knee surgeons, (J.A.F., T.S.W., B.M.D.) at the same clinic using an arthroscopically assisted technique. The femoral and tibial tunnels were drilled anatomically with the femoral tunnel drilled via the anteromedial portal. A fixed suspensory button system was used for femoral fixation (EndoButton; Smith & Nephew Endoscopy), while on the tibial side a cannulated interference screw was used. For the HS technique, a 4-strand graft (doubled semitendinosus/doubled gracilis) was used, attached to an EndoButton CL Ultra device (Smith & Nephew Endoscopy). For the QT technique, a partial thickness soft tissue–only graft was harvested with the smaller-diameter end of the graft being attached to an EndoButton (Smith & Nephew Endoscopy) with nonabsorbable whipstitches. Graft selection was based on a discussion between the surgeon and the patient and their family, when appropriate. The decision-making process took into account factors such as patient preferences, the type of sport to which the patient wished to return, and the patient’s morphological characteristics.

Rehabilitation guidelines were provided to all patients and were the same regardless of graft type. Because of individual insurance policies, patient preferences, and geographic factors, the amount of supervised physical therapy varied between patients, which reflects current practice in Australia. The postoperative rehabilitation protocol has been published previously.^1,2^ Full weightbearing was permitted from the first postoperative day, and knee range of motion exercises were commenced immediately with a focus on regaining full active terminal knee extension. From 3 weeks, stationary cycling, wall squats, forward lunges, and hamstring curls were introduced. From 5 weeks, patients progressed to gymnasium-based exercises including leg press (concentric and eccentric, both double-leg and single-leg), half squats, calf raises, and exercise ball drills for core stability. Open kinetic chain leg extension exercises were allowed after 8 weeks.

Between 10 and 16 weeks, hopping and landing drills were commenced if there was no effusion and adequate quadriceps strength. Running was allowed from 12 weeks provided there was no effusion and adequate progression of hopping and landing drills. Sport-specific drills were introduced from 16 weeks. From 6 months, a graduated return to team training was allowed by the treating surgeon based on absence of effusion, satisfactory range of motion and quadriceps strength, and a stable knee on clinical examination, as well as good control of a single-leg step-down. Clearance to RTS was typically between 9 and 12 months, as determined by the treating surgeon. Patients were instructed to complete at least 4 weeks of unrestricted training before returning to competitive sports and only to do so if they felt confident in their knee function.

### Data Collection Procedures

Patients completed a questionnaire preoperatively that asked them to provide demographic information and grade their level of sport participation from the following categories: sports sometimes, frequent sports, high-level competitive sports, and professional athlete. Patients were also asked to complete the Marx Activity Rating Scale (MARS) to assess the frequency of activities such as running and cutting, deceleration, and pivoting movements.

At 12 months, patients were asked to complete a questionnaire that consisted of the following patient-reported outcome measures (PROMs): the International Knee Documentation Committee Subjective Knee Form, ACL Return to Sport After Injury Scale (ACL-RSI), MARS, and Knee injury and Osteoarthritis Outcome Score Quality of Life (KOOS-QoL) subscale.

**Figure 2.**
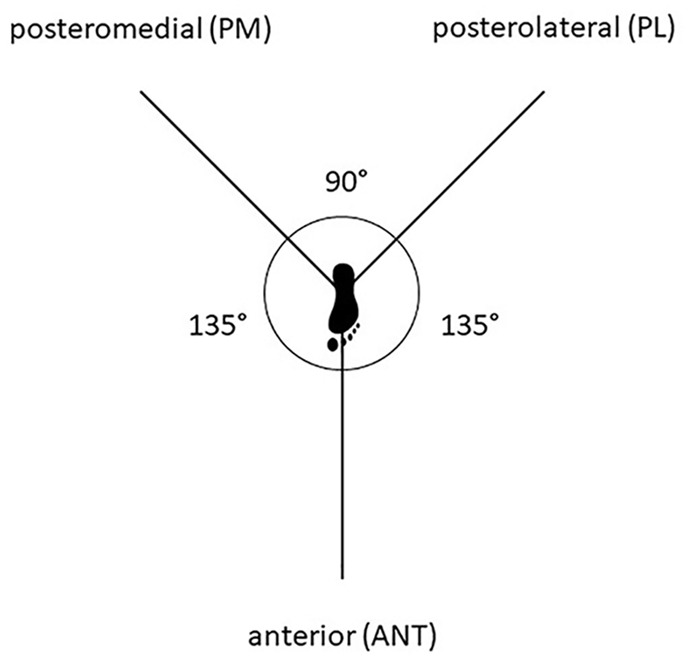
Modified Star Excursion Balance Test setup.

**Figure 3.**
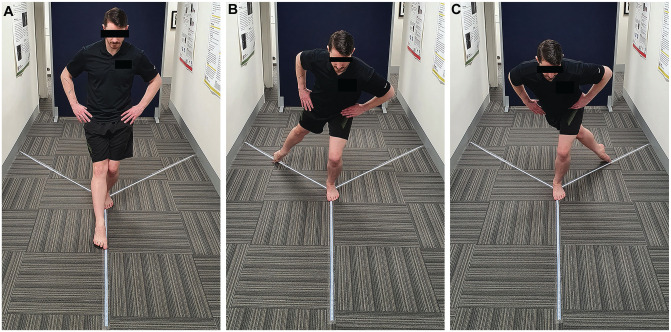
(A) Anterior reach. (B) Posteromedial reach. (C) Posterolateral reach.

A 12-month assessment was also conducted that included a clinical examination of the knee. As part of a battery of strength and functional tests, the MSEBT was performed as follows: Three tape measures with 1-mm marks were adhered to the floor surface in a Y-shape to represent the 3 reach directions (anterior reach 135° apart from posteromedial and posterolateral, which are at a 90° angle to each other) (Figure 2). Patients stood barefoot with 1 foot on the center of the Y formation (Figure 3). While maintaining a single-leg stance, they were instructed to reach with their nonstance lower extremity as far as possible in each direction, gently touch the ground with the great toe to mark the maximum reach, and then return to the starting point under control. The maximum reach distance for each direction was measured in centimeters from the central point of the measuring tape to the point of maximum reach. Patients were instructed to practice on both legs 3 times, before completing 3 recorded attempts. The maximum distance of the 3 recorded attempts was used in analysis. Tests were performed in the following order: right anterior reach, left anterior reach, right posteromedial reach, left posteromedial reach, right posterolateral reach, and left posterolateral reach. The reach distance was expressed as a percentage of leg length: absolute reach distance (cm)/leg length (cm) × 100. The LSI was calculated for each reach direction: reach distance (cm) of affected leg/reach distance (cm) of unaffected leg × 100. In addition, the composite score (CS) was calculated for each limb: CS = [(ANT + PM + PL)/(3 × LL)] × 100, where ANT is anterior, PM is posteromedial, PL is posterolateral, and LL is leg length.^20^

### Statistical Analysis

All statistical analyses were performed using IBM SPSS Statistics Version 28 (IBM Corp) with statistical significance set at a *P* value ≤.05. The Shapiro-Wilk test of normality was used to confirm the normal distribution of all data. Effect sizes were calculated (Cohen *d*) and classified as small (*d* = 0.2), medium (*d* = 0.5), or large (*d* ≥ 0.8). The LSI-MSEBT results, PROMs, age at time of surgery, and anthropometrics were compared between HS and QT ACLR groups using independent-samples *t* tests. The proportion of patients who had an MSEBT-LSI >90 or ≤90 were compared between the HS and QT ACLR groups using chi-square analysis.

## Results

Demographic data of the participants are summarized in Table 1. The only difference between the 2 groups was that the QT cohort was statistically significantly taller than the HS cohort (*P* = .049) and had a higher percentage of patients who documented their sporting level as high-level competitive sport (*P* = .04).

**Table 1:** Patient Characteristics^*a*^

	QT (n = 44)	HS (n = 88)
Age, y	21.75 (4.18)	22.42 (4.38)
Sex, %		
Female	18.2	18.2
Male	81.8	81.8
Preoperative activity level		
Frequent sports	17 (38.64)	51 (57.95)
High-level competitive sports	27 (61.36)	37 (42.05)
Preoperative MARS score	13.82 (3.28)	12.85 (3.16)
Height, cm	180.5 (7.71)	177.6 (8.24)
Weight, kg	81.5 (11.8)	80.3 (13.8)
Proximal graft diameter, mm	8.72 (0.95)	8.11 (0.61)
Distal graft diameter, mm	9.27 (0.80)	8.35 (0.64)
Medial meniscus injury and treatment		
Intact	38 (86.36)	60 (68.18)
Stable tear, no treatment	4 (9.09)	5 (5.68)
Partial meniscectomy	1 (2.27)	11 (12.50)
Previous resection	0 (0.0)	1 (1.14)
Repair	1 (2.27)	11 (12.50)
Lateral meniscus injury and treatment		
Intact	22 (50.00)	55 (62.50)
Stable tear, no treatment	8 (18.18)	12 (13.64)
Partial meniscectomy	9 (20.45)	17 (19.32)
Previous resection	0 (0.0)	1 (1.14)
Repair	5 (11.36)	3 (3.40)
Duration from ACLR to measurements, days	383.84 (38.25)	386.44 (33.13)

a Data are presented as mean (SD) or n (%) unless otherwise indicated. ACLR, anterior cruciate ligament reconstruction; HS, hamstring tendon; MARS, Marx Activity Rating Scale; QT, quadriceps tendon.

There was no significant difference between groups for patient-reported outcomes, which are summarized in Table 2.

**Table 2:** PROMs 12 Months Postoperatively^*a*^

	QT (n = 41)	HS (n = 87)	*P* Value
RTS	13 (29.54)	30 (34.09)	.690
IKDC score	85.98 (12.93)	87.14 (11.49)	.609
ACL-RSI score	70.73 (23.45)	67.17 (24.70)	.441
MARS score	9.76 (4.77)	9.56 (4.36)	.821
KOOS-QoL	72.80 (17.36)	70.99 (15.79)	.557

a Data are presented as mean (SD) or n (%). ACL-RSI, Anterior Cruciate Ligament Return to Sport After Injury Scale; HS, hamstring tendon; IKDC, International Knee Documentation Committee; KOOS-QoL, Knee injury and Osteoarthritis Outcome Score Quality of Life; MARS, Marx Activity Rating Scale; PROM, patient-reported outcome measure; QT, quadriceps tendon; RTS, return to sport.

Table 3 summarizes the MSEBT measurements for both groups. Apart from a small difference in the posterolateral measurements normalized to leg length, there were no statistically significant differences between the QT and HS groups. Effect sizes were negligible to small.

**Table 3:** Summary of Modified Star Excursion Balance Test Measurements^*a*^

	QT (n = 44)	HS (n = 88)	*P* Value	Cohen *d*
Anterior reach				
Operated limb normalized to leg length, %	98.7 (8.98)	98.6 (9.35)	.962	0.009
Nonoperated limb normalized to leg length, %	98.9 (9.11)	99.8 (8.50)	.615	0.093
LSI	99.9 (4.92)	98.9 (5.20)	.309	0.189
% patients with LSI ≥90%	97.73	96.59	.720	
Posterolateral reach				
Operated limb normalized to leg length, %	103.41 (11.17)	99.15 (11.88)	.050	0.366
Nonoperated limb normalized to leg length, %	102.98 (12.97)	99.28 (12.70)	.119	0.290
LSI	100.90 (7.19)	100.16 (6.19)	.540	0.114
% patients with LSI ≥90%	95.45	94.32	.784	
Posteromedial reach				
Operated limb normalized to leg length, %	96.77 (11.54)	92.97 (13.11)	.105	0.302
Nonoperated limb normalized to leg length, %	96.21 (11.71)	92.44 (12.71)	.102	0.305
LSI	101.07 (9.13)	100.75 (6.86)	.824	0.041
% patients with LSI ≥ 90%	93.18	95.45	.583	
Composite scores				
Operated limb	96.63 (9.22)	96.92 (9.53)	.121	0.288
Nonoperated	99.38 (9.54)	97.16 (9.68)	.214	0.231
LSI	100.44 (5.43)	99.86 (4.49)	.517	0.120
% patients with LSI ≥90%	97.73	95.45	.473	

a Data are presented as mean (SD) unless otherwise indicated. HS, hamstring tendon; LSI, limb symmetry index; QT, quadriceps tendon.

## Discussion

The main finding of this study was that there were no differences in our ACLR patient cohort between QT and HS autografts 12 months after ACLR in the anterior and lateral reach distances, the symmetry of reach distances, or the CSs of the MSEBT between graft types. The only difference between patient groups was in the posterolateral reach direction, in which better performance was recorded in the QT cohort (*P* = .05) with a small to moderate effect, which may be clinically relevant. These findings suggest that regardless of graft type, dynamic balance symmetry and performance, at least as measured by MSEBT results, are largely restored 12 months after ACLR. This statement is supported by the fact that the PROMs also show no significant difference at this time.

QT autograft is becoming increasingly popular, although there is a paucity of literature regarding the performance of the MSEBT and YBT in patients who have undergone an ACLR with a QT graft.^6,24,32^ Saper et al^24^ are the only other authors to have investigated dynamic balance in patients with QT autograft. The authors conducted serial YBT assessments at 6 and 9 months and reported a significant improvement in anterior reach results noted over this time frame, improving from <70% achieving anterior reach symmetry within 4 cm of the contralateral limb at 6 months to 95% at 9 months. These findings of symmetry in dynamic balance are similar to those in the current study, where at least 94% of patients achieved an LSI of ≥90% for each reach direction.

Comparison of MSEBT and YBT performance between graft types other than QT and different testing times has previously been explored. In contrast to our study, Sugimoto et al^27^ tested 6 to 9 months postoperatively and demonstrated a significant limb asymmetry for anterior reach of the YBT for the PT graft compared with the HS graft (94.1 vs 99.9 symmetry, respectively). The authors attributed this difference to the likely presence of anterior knee pain in the PT group. This might possibly explain the lack of difference in anterior reach between the groups in the current study, as anterior knee pain seems to be less of an issue in the QT autograft compared with the PT autograft.^15^

In the current study, patients were tested at 12 months, which is substantially longer postoperatively than other reported assessments of dynamic balance after ACLR. It is possible that testing earlier may have demonstrated greater limb asymmetry or a difference between the 2 graft types that was not present at 12 months. A previous study lends support to this possibility: Saper et al^24^ showed improved performance between the 6- and 9-month test results. Thus, further improvement at 12 months could result in a ceiling effect such that no limb asymmetry was apparent.

The aforementioned studies compared the injured limb of athletes with the uninjured limb. Comparison with a control cohort may provide different and valuable information. Delahunt et al^6^ compared dynamic postural stability in a female athlete cohort who underwent ACLR (with PT or HS graft) with a cohort of uninjured athletes using the SEBT. A significant difference was found for the posterolateral and posteromedial reach directions with better performance in the uninjured cohort. However, because no single study has compared dynamic balance between limbs as well as with an uninjured cohort in patients with QT graft, we cannot assume that the lack of asymmetry in the current study is reflective of full recovery of the operated limb rather simply matching worsened dynamic balance performance in the noninjured limb.

Dynamic balance is the ability to maintain balance while the body is moving.^29^ Controlling dynamic balance requires a complex process of collecting and centrally processing sensory information that produces coordinated movement through the musculoskeletal system so that balance is maintained during anticipated movements. It is possible that, given the reliance on central coordination of balance, any interruption to this complex system through unilateral injury will ultimately interrupt balance bilaterally. In support of this, there is some evidence that after ACLR, dynamic balance changes are seen in both the injured and uninjured limbs. Clagg et al^5^ compared 66 patients after ACLR (26 with PT graft, 32 with HS graft, and with 8 allograft) with an uninjured control group at a mean of 6.7 months postoperatively using the MSEBT and found that participants with ACLR had reduced performance for anterior reach in both the injured and uninjured limbs but that there was no significant difference between limbs. Therefore, further research is needed to determine whether the symmetry reported in the current study is related solely to improvements in dynamic balance on the operated limb or whether it also reflects a reduction in performance on the nonoperated limb.

Although achieving symmetry in performance between limbs is a common goal of postoperative rehabilitation, regaining symmetry does not necessarily mean that function and performance are at a preinjury level and, by extension, the risk for further injury is reduced. Although this study did not utilize the MSEBT in the context of an RTS battery of tests, other studies have demonstrated that excellent limb symmetry may not necessarily be associated with a safer RTS. Wellsandt et al^31^ found that the majority of athletes who experienced a second ACL rupture had passed the 90% LSI threshold for strength and hop tests at 6 months postoperatively. Other studies, such as that by Chimera et al^4^ have highlighted the importance of the evaluation of movement patterns on the YBT rather than only the overall scores and symmetry. It is clear that further work is needed to identify the ideal protocol for assessment of balance after ACLR as it relates to predicting a safe RTS.

### Limitations

As previously stated, a limitation of this study is that we have not compared the patients with uninjured controls. This does limit the interpretation and understanding of the high symmetry reported. Also, baseline values before a potential injury, as is increasingly done in preseason testing of professional athletes, would certainly be of extreme importance for correct interpretation of the test results.

A further limitation is that although matched, patients were not randomized to one graft or the other, and there could be factors other than graft type that influenced performance on the MSEBT that we have not controlled for.

## Conclusion

This study found no significant difference for the LSI on the MSEBT between QT and HS autografts with >98% limb symmetry in each reach direction in both groups at 12 months after ACLR.
